# Prevalence of dyslipidemia, treatment rate and its control among patients with type 2 diabetes mellitus in Northwest China: a cross-sectional study

**DOI:** 10.1186/s12944-022-01691-1

**Published:** 2022-08-25

**Authors:** Jiahang Li, Zhenxing Nie, Zhongli Ge, Lei Shi, Bin Gao, Yan Yang

**Affiliations:** 1 Department of Pharmacy, The Second Affiliated Hospital of Air Force Medical University, Xi’an 710038, China; 2 Department of Pharmacy, Hanzhong Municipal People’s Hospital, Hanzhong, 723000 China; 3 Department of Endocrinology, The Second Affiliated Hospital of Air Force Medical University, Xi’an 710038, China

**Keywords:** Dyslipidemia, Prevalence, Treatment, Control, Type 2 diabetes mellitus

## Abstract

**Background:**

The prevalence of cardiovascular disease (CVD) is high in China, especially in Northwest China, and dyslipidemia in diabetes is a major factor at risk for CVD. The dyslipidemia prevalence, treatment and control among type 2 diabetes mellitus (T2DM) patients in Northwest China were investigated.

**Methods:**

In the cross-sectional retrospective research, 1386 medical records of T2DM patients were collected from the Endocrine Department of Tangdu Hospital. And patients’ age, sex, diabetes duration, glycated hemoglobin (HbA1c), complications, lipid levels, and drug use were recorded. The patient characteristics, lipid level and lipid-lowering therapy were analyzed.

**Results:**

In this study, the dyslipidemia prevalence among T2DM patients was 87.7%, the treatment rate was 68.0%. The overall control rate of low-density lipoprotein cholesterol (LDL-C) was 43.1%, and control rates reached 52.7% for high-risk subjects and 36.1% for very high-risk subjects. The overall control rate of non-high-density lipoprotein cholesterol (non-HDL-C) was 19.8%. HbA1c (%) ≥ 7 was indicated as a major factor predicting failure of LDL-C and non-HDL-C control [odds ratio (OR) 1.521; 2.206, 95% confidence interval (CI) 1.154–2.005; 1.583–3.076)].

**Conclusion:**

Among patients with T2DM, it is high prevalence of dyslipidemia and low rate of treatment and control, and higher HbA1c level is the main factor for poor lipid control. It calls for more efforts to promote early screening, prevention and treatment of dyslipidemia for patients, thereby reducing the risk of CVD.

**Supplementary Information:**

The online version contains supplementary material available at 10.1186/s12944-022-01691-1.

## Introduction

Type 2 diabetes mellitus (T2DM) is an abnormality in glucose metabolism and related metabolic disorders caused by inadequate insulin secretion or ineffective cellular response to insulin [1, 2]. Recent survey results indicated there were 537 million adults with DM worldwide, accounting for about 10.5% of the number of people ranging from 20–79 years old. T2DM accounts for over 90% of DM cases and has become one of the chronic diseases that seriously threaten human health [3]. Cardiovascular disease (CVD) dominates the mortality factors in T2DM patients, and approximately 66.3% of patients with diabetes die from CVD [4, 5]. Dyslipidemia and DM can significantly increase the risk of CVD, both are independent risk factors. However, T2DM patients often suffer from dyslipidemia, resulting in a higher risk of CVD and death [6, 7]. Therefore, early screening, timely diagnosis and effective intervention for dyslipidemia are effective strategies to decrease the risk of CVD.

Dyslipidemia affected approximately 70.0%-85.0% of T2DM patients, the prevalence is very high [7]. Diabetic dyslipidemia is particularly severe in developing countries [8]. China is a developing country with the largest population. Chronic diseases are responsible for 88.5% of deaths each year due to the severe ageing of the population. Among them, cardiovascular diseases rank first and have become a serious public health problem. The geography and natural environment of Northwest China have resulted in a diet that is mostly high in carbohydrates and fats, with few vegetables and fruits. Therefore, T2DM and dyslipidemia are widely prevalent in this area, leading to a high prevalence of CVD [9]. Nevertheless, in recent years, the data related to diabetic dyslipidemia is scarce in this region.

In this study, the prevalence, treatment and control of dyslipidemia and risk factors were analyzed in patients with T2DM to provide an effective reference for the management of dyslipidemia.

## Methods

### Patient population

**Fig. 1:**
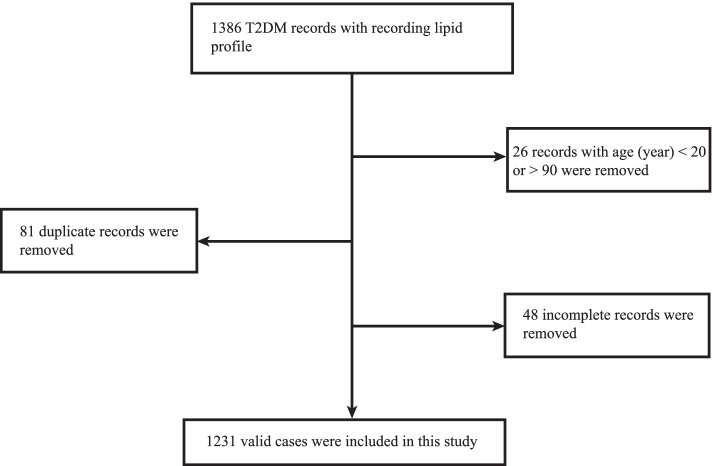
Flowchart of population selection

This is a cross-sectional retrospective study. The researchers collected 1386 T2DM patients’ data at Tangdu Hospital from March 2021 to February 2022 through the medical record system. The patients were mainly from the northwestern region in China such as Shaanxi, Gansu, Qinghai, Ningxia, and Xinjiang province. The valid cases included (1) subjects with age between 20–90 years; (2) clear T2DM history; (3) recording lipid profile. Excluding age-ineligible medical data (26), duplicate data (81) and incomplete data (48), the number of valid cases is 1231 (Fig. 1).

### Data collection

Three researchers had undergone rigorous training and assessment before they collected the data. They used a case report form to collect data from the patients’ medical records. The case report form contained body mass index (BMI), age, sex, DM duration, lipid-lowering medications, glycated hemoglobin (HbA1c) level, serum lipid level and concomitant diseases. The concomitant diseases included atherosclerotic cardiovascular disease (ASCVD), hypertension, diabetic kidney disease (DKD), diabetic peripheral neuropathy (DPN), diabetic retinopathy (DR) and fatty liver disease (FLD).

### Risk classification and diagnostic criteria

The diagnostic criteria for T2DM was derived from the World Health Organization’s [3]. The definition of dyslipidemia refered to the “Chinese Guidelines for the Prevention and Treatment of Dyslipidemia in Adults” [10]. According to the guidelines, the patients enrolled were evaluated for dyslipidemia. Serum triglycerides (TG) ≥ 2.26 mmol/L, serum total cholesterol (TC) ≥ 6.22 mmol/L, serum high-density lipoprotein cholesterol (HDL-C) < 1.04 mmol/L and serum low-density lipoprotein cholesterol (LDL-C) ≥ 4.14 mmol/L, one of the above indexes or diagnosed by the hospital and currently taking lipid-lowering drugs, which is treated as dyslipidemia. By the patient’s serum lipid level and medication, the dyslipidemia patients included the treatment subjects and the non-treatment subjects with dyslipidemia. Elevated or decreased lipid profiles were based on the above cutoff values. Patients were assessed for cardiovascular risk in accordance with the “Clinical guidelines for prevention and treatment of type 2 diabetes mellitus in the elderly in China (2022 edition)”. Those with a defined record of ASCVD were classified as very high-risk and the rest as high-risk [3]. The target of LDL-C management is as follows: < 2.6 mmol/L (high-risk) or < 1.8 mmol/L (very high-risk). The target for non-HDL-C is as follows: < 2.6 mmol/L (high-risk) or < 2.2 mmol/L (very high-risk). Other comprehensive control goals include HbA1c (%) < 7, blood pressure (mmHg) < 130/80, BMI (kg/m^2^) < 24, HDL-C (mmol/L) > 1.3 for female or > 1.0 for male, TC (mmol/L) < 4.5 and TG (mmol/L) < 1.7 [3].

### Statistical analysis

In this research, all data were analyzed statistically in SPSS Statistics 26. Values were summarized as the mean ± standard deviation (SD) or frequency (percentage). Chi-square test or Fisher's exact test and independent samples t-test for comparisons of categorical and continuous variables were conducted respectively. To investigate the effects of sex, age, DM duration, HbA1c, BMI, hypertension and FLD on non-treatment and non-attainment of dyslipidemia, multiple logistic regression analysis was performed. A two-sided *P *value of < 0.05 was interpreted as statistically significant.

## Results

### Patient characteristics

All population characteristics are represented in Table 1. There were 1231 patients, of which 476 were over 60 years old and 838 were men. The mean percentage of HbA1c (%) was 8.57 ± 2.20, with 338 patients HbA1c less than 7%. The average duration of T2DM was 9.30 ± 7.13 years, with 465 patients over 10 years. The mean BMI index was 25.96 ± 3.68 kg/m^2^, and the number of patients less than 24 kg/m^2^ was 365. Complications of ASCVD, DKD, DR and DPN in this population accounted for 37.2%, 34.6%, 22.3%, and 29.2%, respectively, and patients with hypertension and FLD accounted for 47.0% and 24.9%, respectively. Meanwhile, the mean serum lipid level was 4.42 ± 1.17 mmol/L for TC, 2.03 ± 1.82 mmol/L for TG, 2.48 ± 0.94 mmol/L for LDL-C, and 1.04 ± 0.29 mmol/L for HDL-C.

**Table 1:** The patient characteristics

	**All patients (*N* = 1231)**
Male	838 (68.1%)
Age (years)	55.74 ± 13.23
> 50	845 (68.6%)
> 60	476 (38.7%)
HbA1c (%)	8.57 ± 2.20
< 7	338 (27.5%)
T2DM duration (years)	9.30 ± 7.13
> 10	465 (37.8%)
BMI(kg/m^2^)	25.96 ± 3.68
< 24	365 (29.7%)
ASCVD	458 (37.2%)
DKD	426 (34.6%)
DR	274 (22.3%)
DPN	359 (29.2%)
Hypertension	579 (47.0%)
FLD	306 (24.9%)
TC (mmol/L)	4.42 ± 1.17
TG (mmol/L)	2.03 ± 1.82
LDL-C (mmol/L)	2.48 ± 0.94
HDL-C (mmol/L)	1.04 ± 0.29

*Abbreviations*: *ASCVD* Atherosclerotic cardiovascular disease, *BMI* Body mass index, *DR* Diabetic retinopathy, *DPN* Diabetic peripheral neuropathy, *DKD* Diabetic kidney disease, *FLD* Fatty liver disease, *HbA1c* Glycated hemoglobin, *HDL-C* High-density lipoprotein cholesterol, *LDL-C* Low-density lipoprotein cholesterol, *TC* Total cholesterol, *TG* Triglycerides

### Dyslipidemia rate

The rate of dyslipidemia and the population characteristics are shown in Table 2. In this study, there were only 152 patients with normal lipids, accounting for 12.3%. While there were 1079 patients with dyslipidemia, and the dyslipidemia rate was 87.7%. Patients with > 60 years old, HbA1c level of 7%-10%, T2DM duration ≥ 10 years, and BMI (kg/m^2^) ≥ 24 had the lowest normolipidemia rate, which were 10.3%, 9.2%, 11.2%, and 8.9%, respectively. In the non-hypertensive and non-FLD patients, the lipid normolipidemia rates were 13.8% and 14.3%, significantly higher compared with the hypertensive and FLD patients.

**Table 2:** Dyslipidemia rate and the characteristics

	**Normolipidemia**	**Dyslipidemia**		***P* value**
		**Non-treatment**	**Treatment**	
N (%)	152 (12.3%)	345 (28.1%)	734 (59.6%)	< 0.0001
Sex				
male	92 (11.0%)	246 (29.4%)	500 (59.7%)	
female	60 (15.3%)	99 (25.2%)	234 (59.5%)	0.0600
Age (years)				
< 50	46 (12.5%)	189 (51.4%)	133 (36.1%)	
50–60	57 (14.7%)	90 (23.3%)	240 (62.0%)	
> 60	49 (10.3%)	66 (13.9%)	361 (75.8%)	< 0.0001
HbA1c (%)				
< 7	49 (14.5%)	82 (24.3%)	207 (61.2%)	
7–10	53 (9.2%)	153 (26.5%)	372 (64.4%)	
> 10	50 (15.9%)	110 (34.9%)	155 (49.2%)	< 0.0001
T2DM duration (years)				
< 3	33 (11.7%)	125 (44.5%)	123 (43.8%)	
3–10	67 (13.8%)	151 (31.1%)	267 (55.1%)	
> 10	52 (11.2%)	69 (14.8%)	344 (74.0%)	< 0.0001
BMI (kg/m^2^)				
< 24	75 (20.5%)	74 (20.3%)	216 (59.2%)	
≥ 24	77 (8.9%)	271 (31.3%)	518 (59.8%)	< 0.0001
Hypertension				
Yes	62 (10.7%)	108 (18.7%)	409 (70.6%)	
No	90 (13.8%)	237 (36.3%)	325 (49.8%)	< 0.0001
FLD				
Yes	20 (6.5%)	97 (31.7%)	189 (61.8%)	
No	132 (14.3%)	248 (26.8%)	545 (58.9%)	0.0010

Values were frequency (percentage). *P* value was calculated by Chi-square test or Fisher’s exact test

### Pattern of dyslipidemia

**Fig. 2:**
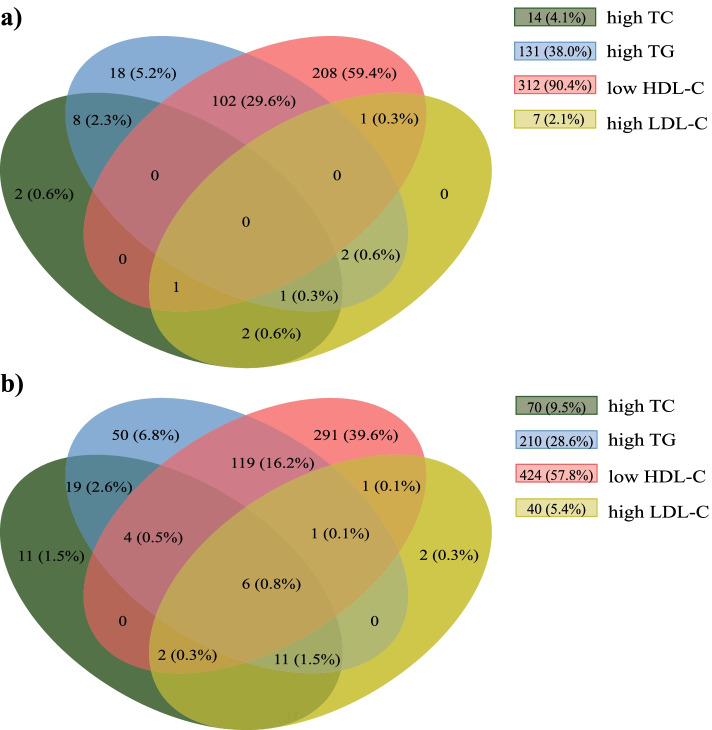
Distribution of the dyslipidemia pattern. **a** in the non-treatment patients, **b** in the treatment patients

The distribution of the pattern of dyslipidemia is shown in Fig. 2. Low HDL-C and high TG contributed to the major dyslipidemia patterns. In the non-treatment subjects (Fig. 2A), low HDL-C and high TG were in 90.4% and 38.0% of patients, respectively. 29.6% of patients showed low HDL-C + high TG. In the treatment subjects (Fig. 2B), high TG (28.6%) and low HDL-C (57.8%) were the common patterns of dyslipidemia. Moreover, low HDL-C + high TG accounted for 16.2% of treatment subjects.

### Treatment rate and the lipid-lowering treatment

Treatment rate and treatment population characteristics are shown in Table 3. There were 1079 T2DM patients with dyslipidemia, of which 68.0% (734) patients received lipid-lowering treatment and 32.0% (345) patients did not receive lipid-lowering treatment. In the treatment group, 92.6% of patients were on statins, 6.8% were on fibrates and only 0.6% were on a combination of statins and fibrates. Atorvastatin and rosuvastatin were the most frequently used medications, by 59.6% and 28.0% of the patients, respectively, compared with 12.4% for other statins.The rate of treatment increased with the age, HbA1c level, BMI, duration of T2DM and the onset of other diseases (hypertension, ASCVD, DKD, DR, DPN). After treatment, the mean HDL-C level increased significantly, while there were no significant changes in the other measures of the lipid profiles.

**Table 3:** Treatment rate and the characteristics

	**Non-treatment**	**Treatment**	***P* value**
N (%)	345 (32.0%)	734 (68.0%)	< 0.0001
Sex			
male	246 (33.0%)	500 (67.0%)	
female	99 (29.7%)	234 (70.3%)	0.2910
Age (years)			
< 50	189 (58.7%)	133 (41.3%)	
50–60	90 (27.3%)	240 (72.7%)	
> 60	66 (15.5%)	361 (84.5%)	< 0.0001
HbA1c (%)			
< 7	82 (28.4%)	207 (71.6%)	
7–10	153 (29.1%)	372 (70.9%)	
> 10	110 (41.5%)	155 (58.5%)	0.0010
T2DM duration (years)			
< 3	125 (50.4%)	123 (49.6%)	
3–10	151 (36.1%)	267 (63.9%)	
> 10	69 (16.7%)	344 (83.3%)	< 0.0001
BMI (kg/m^2^)			
< 24	74 (25.5%)	216 (74.5%)	
≥ 24	271 (34.3%)	518 (65.7%)	0.0060
Hypertension			
Yes	108 (20.9%)	409 (79.1%)	
No	237 (42.2%)	325 (57.8%)	< 0.0001
FLD			
Yes	97 (33.9%)	189 (66.1%)	
No	248 (31.3%)	545 (68.7%)	0.4110
ASCVD			
Yes	33 (7.5%)	407 (92.5%)	
No	312 (48.8%)	327 (51.2%)	< 0.0001
DKD			
Yes	84 (22.6%)	288 (77.4%)	
No	261 (36.9%)	446 (63.1%)	< 0.0001
DR			
Yes	46 (19.4%)	191 (80.6%)	
No	299 (35.5%)	543 (64.5%)	< 0.0001
DPN			
Yes	56 (17.6%)	263 (82.4%)	
No	289 (38.0%)	471 (62.0%)	< 0.0001
Lipid profile (mmol/L)			
TC	4.32 ± 0.97	4.44 ± 1.31	0.0940
TG	2.20 ± 1.66	2.13 ± 2.01	0.5750
HDL-C	0.90 ± 0.17	1.05 ± 0.29	< 0.0001
LDL-C	2.52 ± 0.80	2.44 ± 1.05	0.1530

Values were frequency (percentage) or mean + SD. *P* value was calculated by Chi-square test or Fisher's exact test and independent samples t-test

### Lipid goal attainment

The lipid control is described in Table 4. 43.1% of treatment subjects attained the LDL-C goal, specifically 52.7% for the high-risk and 36.1% for the very high-risk. The LDL-C control rate increased progressively with age, duration of T2DM, and onset of hypertension and decreased with increasing HbA1c levels and onset of FLD. The overall non-HDL-C control rate was 19.8%, it was 19.9% and 19.7% in high-risk and very high-risk patients. The characteristics of the patients attaining non-HDL-C goal were essentially similar to those attaining LDL-C goal.

**Table 4:** Lipid control for the treatment

	**LDL-C control**		**Non-HDL-C control**	
	**High-risk (327)**	**Very high-risk (407)**	**High-risk (327)**	**Very high-risk(407)**
N (%)	169 (51.7%)	147 (36.1%)	65 (19.9%)	80 (19.7%)
*P* value	0.5800	< 0.0001	< 0.0001	< 0.0001
Sex				
female	44 (44.9%)	49 (36.0%)	16 (16.3%)	24 (17.6%)
male	125 (54.6%)	98 (36.2%)	49 (21.4%)	56 (20.7%)
*P* value	0.1080	0.9790	0.2930	0.4700
Age (years)				
< 50	41 (42.7%)	8 (21.6%)	6 (6.3%)	4 (10.8%)
50–60	52 (46.0%)	39 (30.6%)	23 (20.4%)	18 (14.2%)
> 60	76 (64.4%)	100 (41.2%)	36 (30.5%)	58 (23.9%)
*P* value	0.0020	0.0220	< 0.0001	0.0300
HbA1c (%)				
< 7	62 (68.1%)	48 (41.4%)	35 (38.5%)	30 (25.9%)
7–10	84 (50.6%)	72 (35.0%)	24 (14.5%)	40 (19.4%)
> 10	23 (32.9%)	27 (31.8%)	6 (8.6%)	10 (11.8.%)
*P* value	< 0.0001	0.3310	< 0.0001	0.0450
T2DM duration (years)				
< 3	27 (38.0%)	14 (26.9%)	6 (8.5%)	6 (11.5%)
3–10	69 (53.1%)	59 (43.1%)	27 (20.8%)	34 (24.8%)
> 10	73 (57.9%)	74 (33.9%)	32 (25.4%)	40 (18.3%)
*P* value	0.0250	0.0740	0.0160	0.0950
BMI (kg/m^2^)				
< 24	46 (51.1%)	49 (38.9%)	21 (23.3%)	27 (21.4%)
≥ 24	123 (51.9%)	98 (34.9%)	44 (18.6%)	53 (18.9%)
*P* value	0.8990	0.4360	0.3350	0.5470
Hypertension				
Yes	99 (62.7%)	103 (41.0%)	46 (29.1%)	59 (23.5%)
No	70 (41.4%)	44 (28.2%)	19 (11.2%)	21 (13.5%)
*P *value	< 0.0001	0.0090	< 0.0001	0.0130
FLD				
Yes	39 (42.9%)	29 (29.6%)	10 (11.0%)	11 (11.2%)
No	130 (55.1%)	118 (38.2%)	55 (23.3%)	69 (22.3%)
*P* value	0.0470	0.1230	0.0120	0.0160

Values were frequency (percentage). *P* value was calculated by Chi-square test or Fisher’s exact test

### Dyslipidemia rate in patients with complications

Common complications of diabetes include ASCVD, DKD, DR and DPN, and the number of patients with complications was 407, 288, 191, and 263, respectively. Non-HDL-C levels failed to reach target values in 76.0% to 83.0% of patients, and it was the highest in DKD patients. The rate of LDL-C not at goal ranged from 54.8% to 63.9%, with 63.9% in ASCVD patients (Table 5).

**Table 5:** Dyslipidemia rate in patients with complications

	**ASCVD (407)**	**DKD (288)**	**DR (191)**	**DPN (263)**
TC	143 (35.1%)	139 (48.3%)	80 (41.9%)	94 (35.7%)
TG	151 (37.1%)	151 (52.4%)	76 (39.8%)	88 (33.5%)
HDL-C	278 (68.3%)	170 (59.0%)	113 (59.2%)	165 (62.7%)
LDL-C	260 (63.9%)	174 (60.4%)	109 (57.1%)	144 (54.8%)
Non-HDL-C	327 (80.3%)	239 (83.0%)	145 (76.0%)	209 (79.5%)

### Multivariate risk assessment for non-treatment and non-attainment

Multivariate logistic regression result demonstrated that various factors associated with dyslipidemia non-treatment (Table 6). The age ≤ 60 years (OR 3.124, 95% CI 2.250–4.337), BMI ≥ 24 (OR 1.669, 95% CI 1.192–2.336) and duration of T2DM ≤ 3 years (OR 1.995, 95% CI 1.478–2.694) were significantly associated with an increased risk of non-treatment. The FLD (OR 0.695, 95% CI 0.502–0.961) and hypertension (OR 0.488, 95% CI 0.364–0.655) showed a significant correlation with a decreased risk of non-treatment. Moreover, there were significant correlations between LDL-C and non-HDL-C treatment failure and multiple variables (Table 6), including age ≤ 60 years (OR 1.363; 1.912, 95% CI 1.041–2.786; 1.357–2.693), hypertension (OR 0.762;0.687, 95% CI 0.589–0.985; 0.490–0.964) and HbA1c (%) ≥ 7 (OR1.521; 2.206, 95% CI 1.154–2.005; 1.583–3.076).

**Table 6:** Multivariate risk assessment for non-treatment and non-attainment

Variables	Non-treatment		LDL-C not at goal		Non-HDL-C not at goal	
	**OR (95% CI)**	***P* value**	**OR (95% CI)**	***P* value**	**OR (95% CI)**	***P* value**
Female	1.176 (0.862–1.604)	0.3080	1.226 (0.935–1.607)	0.1400	1.293 (0.904–1.851)	0.1590
Age (years) ≤ 60	3.124 (2.250–4.337)	< 0.0001	1.363 (1.041–2.786)	0.0240	1.912 (1.357–2.693)	< 0.0001
HbA1c (%) ≥7	1.043 (0.758–1.436)	0.7960	1.521 (1.154–2.005)	0.0030	2.206 (1.583–3.076)	< 0.0001
T2DM duration (years) ≤ 3	1.995 (1.478–2.694)	< 0.0001	0.952 (0.717–1.650)	0.7340	1.228 (0.822–1.835)	0.3160
BMI (kg/m^2^) ≥ 24	1.669 (1.192–2.336)	0.0030	1.033 (0.777–1.373)	0.8230	1.213 (0.850–1.731)	0.2870
Hypertension	0.488 (0.364–0.655)	< 0.0001	0.762 (0.589–0.985)	0.0380	0.687 (0.490–0.964)	0.0300
FLD	0.695 (0.502–0.961)	0.0280	1.232 (0.921–1.647)	0.1610	1.968 (1.259–3.086)	0.0030

*Abbreviations*: *OR* Odds ratio, *CI* Confidence interval

## Discussion

The survey research showed that the dyslipidemia prevalence among T2DM patients was 87.7%, the treatment rate was 68.0%, the overall control rates for LDL-C and non-HDL-C were 43.1% and 19.8% in Northwest China.

Dyslipidemia occurs very frequently in T2DM, influencing approximately 70.0% to 85.0% of patients [7, 8]. In this study, the dyslipidemia rate was 87.7%. The dyslipidemia rate in India was 85.5%-97.8% [11], and in Spain it was 81.2% [12], which is similar to this finding. While a study in North and East China showed dyslipidemia rate of 67.1% [6], which is lower than the dyslipidemia rate in this survey. Due to the high carbohydrate, high fat, low vegetable and low fruit diet; lack of exercise; and relatively poor economic level and medical conditions, the dyslipidemia rate is higher in Northwest China. In addition, the patient’s emphasis on self-health also affects the dyslipidemia rate. Moreover, high TG and low HDL-C contributed to the predominant dyslipidemia patterns in the subjects. Recent studies demonstrated that the common patterns of dyslipidemia included abnormal TG and HDL-C levels in patients with T2DM [13, 14]. In T2DM, insulin resistance and hyperglycemia induce elevated triglycerides, which leads to overproduction of glycerol-rich lipoproteins by the liver. The increase of triglyceride-rich lipoproteins generally correlates to a reduction in high-density lipoproteins and an addition in low-density lipoproteins [14, 15]. In turn, high TG and low HDL-C can lead to insulin resistance, resulting in poor glycemic control, which then creates a vicious cycle.

After lifestyle interventions, early medical treatment is the major approach to regulate dyslipidemia, and statins are recommended as the preferred lipid-lowering drugs according to the guidelines [3, 16]. This survey showed that 68.0% of T2DM patients with dyslipidemia were treated with lipid-lowering drugs, with a significant improvement in HDL-C level. A study in Korea showed that the treatment rate for DM was 26.9% [17], which decreased significantly compared with the results of this study. A 2015 survey in China revealed a 55.9% treatment rate among T2DM patients with dyslipidemia [6]. In the current study, the treatment rate for dyslipidemia reached 68.0%, indicating that China's attention to dyslipidemia has improved. For the lipid-lowering therapy, atorvastatin and rosuvastatin were the main drugs in this study, and the reasons are mainly related to the drug safety and efficacy [18, 19]. Although the harm of dyslipidemia is very serious, 32.0% of the patients were still untreated. Regression analysis found that non-treatment is associated with factors such as age ≤ 60 years, duration of T2DM ≤ 3 years, BMI ≥ 24, hypertension and FLD. The patients were in relatively good physical condition leading to neglect of the severity of the disease, as also demonstrated by the analysis of lipid control.

Lowering LDL-C levels is considered the primary goal for lipid management due to the significant reduction in CVD and mortality risk in DM patients, and lowering non-HDL-C levels is a secondary goal [3, 16]. This study indicated that the LDL-C control rate reached 43.1% overall, it was 51.7% for the high-risk and 36.1% for the very high-risk. However, only 19.8% of patients attained the non-HDL-C goal. An analysis in Europe and Canada showed that the TC and LDL-C control rates were 48.1% and 54.7% in the statin-using DM patients [20]. According to a survey in China by Li Yan in 2011, after treatment with lipid-lowering drugs, about 39.4% of the T2DM patients reduced the LDL-C level to below 2.6 mmol/L, and in very high-risk patients the control rate was 15.3% [6]. Compared with studies in developed countries such as Europe and Canada, the control rate of LDL-C in this present study is lower. However, compared to the results from Li Yan et al., the LDL-C control rate of this population is improved. Microvascular and macrovascular disease are common complications of T2DM. In people with complications, lipid control should be stricter in order to prevent further deterioration of the disease. However, the rate of non-HDL-C not at goal ranged from 76.0% to 83.0%, and it reached 83.0% for DKD patients. The rate of LDL-C non-attainment ranged from 54.8% to 63.9%, with the highest rate in patients with ASCVD. This result is particularly noteworthy for physicians.

The failure to achieve lipid control despite medical intervention is associated with various factors. The regression analysis found that the factors included HbA1c, age and hypertension in this study. Of these, HbA1c was the most relevant factor. Dyslipidemia has been suggested to have a linear relationship with HbA1c in recent studies, and HbA1c could reflect cholesterol and LDL levels among T2DM patients. A marked increase for TC and TG levels and a decrease for HDL-C levels compared to patients with good glycemic control [21–23]. For most patients with DM, the better the glycemic control, the more likely they are to exhibit a more active and healthy lifestyle that leads to better management of lipid levels [17]. The “healthy adherer-effect” [24, 25] seems to explain why the patients in this study with poor HbA1c control had lower rates of lipid control. Advanced age and high blood pressure have always been considered unfavorable factors for lipid control. However, this research found age > 60 years old and hypertension are beneficial to the treatment and lipid control. This may be related to the fact that patients who are older and in poorer physical condition are more aware of their disease and show a better treatment adherence, which leads to better lipid control. This phenomenon may be explained with “health belief model”, a theory which suggests that people believing they are sicker will adopt a healthier approach to decelerate disease progression [17].

One thousand two hundred thirty-one Chinese T2DM patient medical data were analyzed in this study. It is worth noting that the dyslipidemia prevalence in T2DM patients was 87.7%, the treatment rate was 68.0%, and the overall LDL-C and non-HDL-C control rates were 43.1% and 19.8%, respectively. Dyslipidemia should be given more attention from the public health perspective. First, early monitoring dyslipidemia is the basis for effective prevention of ASCVD. Second, lifestyle intervention is a fundamental measure for the treatment of dyslipidemia. In addition, individualised medication protocols have become the current trend in lipid intervention.

### Study strength and limitation

This cross-sectional study provides the latest data on the prevalence, treatment rate and control rate of dyslipidemia among T2DM patients. This result may provide an effective therapeutic strategy for reducing CVD in patients with T2DM. Some limitations remain in this study. The cross-sectional design only can evaluate the relationship between dyslipidemia and the risk factors, and can not assess their exact causal effect. Moreover, this single-centre retrospective study had a small sample selection and number, resulting in the sample choice bias. In addition, there are no clear diagnostic criteria for dyslipidemia in patients with DM, the dyslipidemia prevalence may be lower.

## Conclusion

It is evident that high prevalence, low treatment rate and low control rate are the main characteristics of dyslipidemia in patients with T2DM, and the higher HbA1c level is the main factor for poor lipid control. It calls for more efforts to promote early screening, prevention and treatment of dyslipidemia for T2DM patients, thereby reducing the risk of CVD.

## Supplementary Information

**Additional file 1.**

## Data Availability

All data generated or analyzed during this study is included in this published article.

## Abbreviations

ASCVD: Atherosclerotic cardiovascular disease
CVD: Cardiovascular disease
BMI: Body mass index
CI: Confidence interval
DKD: Diabetic kidney disease
DR: Diabetic retinopathy
DM: Diabetes mellitus
DPN: Diabetic peripheral neuropathy
HbA1c: Glycated hemoglobin
HDL-C: High-density lipoprotein cholesterol
FLD: Fatty liver disease
LDL-C: Low-density lipoprotein cholesterol
TG: Triglycerides
T2DM: Type 2 diabetes mellitus
TC: Total cholesterol
OR: Odds ratio
